# The Adhesion G-Protein-Coupled Receptor, GPR56/ADGRG1, Inhibits Cell–Extracellular Matrix Signaling to Prevent Metastatic Melanoma Growth

**DOI:** 10.3389/fonc.2018.00008

**Published:** 2018-02-01

**Authors:** Michelle W. Millar, Nancy Corson, Lei Xu

**Affiliations:** ^1^Department of Biomedical Genetics, University of Rochester Medical Center, Rochester, NY, United States

**Keywords:** adhesion G-protein-coupled receptors, GPR56, extracellular matrix, melanoma, metastasis

## Abstract

Metastatic growth is considered a rate-limiting step in cancer progression, and upregulation of extracellular matrix (ECM) deposition and cell–ECM signaling are major drivers of this process. Mechanisms to reverse ECM upregulation in cancer could potentially facilitate its prevention and treatment but they are poorly understood. We previously reported that the adhesion G-protein-coupled receptor GPR56/ADGRG1 is downregulated in melanoma metastases. Its re-expression inhibited melanoma growth and metastasis and reduced the deposition of fibronectin, a major ECM component. We hypothesize that its effect on fibronectin deposition contributes to its inhibitory role on metastatic growth. To test this, we investigated the function of GPR56 on cell–fibronectin adhesion and its relationship with metastatic growth in melanoma. Our results reveal that GPR56 inhibits melanoma metastatic growth by impeding the expansion of micrometastases to macrometastases. Meanwhile, we present evidence that GPR56 inhibits fibronectin deposition and its downstream signaling, such as phosphorylation of focal adhesion kinase (FAK), during this process. Administration of the FAK inhibitor Y15 perturbed the proliferation of melanoma metastases, supporting a causative link between the cell adhesion defect induced by GPR56 and its inhibition of metastatic growth. Taken together, our results suggest that GPR56 in melanoma metastases inhibits ECM accumulation and adhesion, which contributes to its negative effects on metastatic growth.

## Introduction

Metastatic disease is the most deadly aspect of cancer progression, yet there are few effective therapies targeting this process specifically (1, 2). Metastasis is a complex process requiring a deep understanding for developing successful treatment strategies. Cancer cells must complete five steps to metastasize to a secondary site: (1) detachment from the primary tumor, (2) intravasation into the circulation, (3) survival in the circulation, (4) extravasation into the distant organ, and (5) survival and growth at the secondary site (hereafter defined as *metastatic growth*). In the metastatic growth step, solitary cells proliferate to form small tumors called micrometastases, and after persistent growth the tumors develop vascular networks to promote their development into macrometastases (3). This is a rate-limiting step in metastasis because cells are often able to survive circulation and seed in distant organs, but are unable to proliferate into metastases (1). Metastasis is a difficult and inefficient process, so cells that are able to survive and thrive in the new environment as metastases are especially difficult to eliminate. One needs a complete understanding of this process to specifically treat the more virulent metastases.

Metastatic growth requires a supportive microenvironment, and when this is not present cancer cells can either remain dormant or die (2, 4). An important part of this cell microenvironment is the extracellular matrix (ECM). The ECM is a network of macromolecules between cells that forms a scaffold to support tissue structure, as well as retain moisture and growth factors necessary for cell survival and proliferation (3, 5, 6). ECM proteins also bind adhesion receptors on cells to activate downstream signaling and modulate cell behavior (7, 8). These functions are thought to promote tumor growth, and ECM has indeed been found elevated in numerous cancer types (5, 9–11). Targeting ECM-mediated signaling may, thus, have potential benefits for diagnosis and treatment of metastatic disease, but whether this is the case remains an open question.

One strategy to target ECM and its mediated signaling is to induce its removal. Removal of ECM is governed mainly by endocytosis of adhesion receptors. Integrins are typical cell–ECM adhesion receptors and their endocytosis has been shown to remove ECM proteins from the matrix, but the effects of this internalization on cancer progression are unknown (12–14). A newly described class of adhesion receptors, adhesion G-protein-coupled receptors (GPCRs), contain ECM adhesion motifs at their extracellular stalks and seven transmembrane domains at their C-termini (15). They are expected to regulate cell adhesion through cell–ECM interaction and G-protein-coupled signaling, and several have been implicated in cancer progression (16). Our lab has previously reported that an adhesion GPCR, GPR56 (ADGRG1), is downregulated in metastatic melanoma and inhibits the growth and metastasis of the human melanoma cell line, MC-1 (17). GPR56 expression also led to decreased fibronectin deposition and cell–ECM signaling in subcutaneous tumors from MC-1 cells (18). We show here that GPR56 inhibits metastatic growth from multiple cell lines with different mutation states, sequestering secondary tumors at the micrometastatic state. Our further analyses reveal that GPR56 inhibits cell adhesion and fibronectin deposition *in vitro* and *in vivo*, and that this inhibition likely contributes to its impediment on metastasis proliferation. Taken together, our data suggest that the loss of GPR56 in human melanomas might result in elevated cell–ECM signaling and ECM accumulation to promote metastatic growth.

## Materials and Methods

### Mice

The NOD scid gamma (NSG) mice (NOD.Cg-*Prkdcscid Il2rgtm1Wjl*/SzJ) and the *Tyr:CreER*; *BRaf^CA^*; *Pten^lox/lox^* (CBP) mice (B6.Cg-*Braf^tm1Mmcm^Pten^tm1Hwu^* Tg(Tyr-cre/ERT2)13Bos/BosJ) (19) were purchased from the Jackson Laboratory (Bar Harbor, ME, USA). *RAG2^−/−^* mice were obtained from Dr. Richard Hynes’ laboratory (Massachusetts Institute of Technology) (18). The CBP mice were crossed with *Gpr56^−/−^* mice (20, 21) (Genentech Inc., San Francisco, CA, USA) to obtain the *Tyr:CreER*; *BRaf^CA/+^*; *Pten^lox/lox^; Gpr56^−/−^* (CBPG) compound strain. CBPG mice were genotyped using the AccuStart II Mouse Genotyping Kit (#76047-138, QuantaBio). All mice were housed in the animal facility at the University of Rochester Medical Center (Rochester, NY, USA). This study was carried out in accordance with the recommendations of the animal care guidelines from the Division of Laboratory Animal Medicine at the University of Rochester Medical Center. The protocol was approved by the University Committee on Animal Resources (UCAR) at the University of Rochester Medical Center.

### Cell Lines

The WM983BR and 451LUCS-BR cell lines were received from Wistar Institute, Philadelphia, PA, USA. The MeWo cell line was purchased from ATCC (HTB-65). The SK-MEL-147 cell line was received from Memorial Sloan Kettering Cancer Center, Basking Ridge, NJ, USA. MC-1 cells were cultured in DMEM (4,500 mg/L glucose), 10% FBS, 200 mM glutamine, pen/strep. MeWo cells were cultured in MEM (1,000 mg/L glucose), 10% FBS, 1.5 g/L NaHCO_3_, NEAA (non-essential amino acids), pen/strep. WM983BR and 451LUCS-BR cells were cultured in DMEM, 5% FBS, 200 mM glutamine, pen/strep, 1 mM SB590885 (Tocris Bioscience, Bristol, UK). SK-MEL-147 cells were cultured in RPMI (2,000 mg/L glucose), 7.5% FBS, 100 mM glutamine, pen/strep.

### Knockdown and Overexpressing Cell Lines

shRNAs for knocking down GPR56 in human melanoma cell lines as well as the control-shRNAs were used as described previously (17). The targeted sequences were KD1, 5′-GGTTAATTCTGTCCAACAA-3′; KD2, 5′-TGCAGGAGTCAGCGTTCAA-3′; CTL1 (targets firefly luciferase), 5′-CGTACGCGGAATACTTCGA-3′ (22); CTL2 (targets renilla luciferase), 5′-GTAGCGCGGTGTATTATAC-3′ (23). Cells were infected with the virus and stable cell lines were selected under puromycin. Empty vector (EV) and GPR56 expressing constructs were used as described previously (18).

### Tumor Induction in CBP Transgenic Mice

Tumors were induced based on the published protocol (19), with some modifications. Six- to eight-week-old CBP or CBPG mice were shaved on the rear left flank and treated with 1 μL of 5 mM 4-hydroxytamoxifen (4-HT) (H7904, Sigma) to induce Cre expression in melanocytes. Tumor growth was monitored by measuring the diameter of the tumors three times weekly, and tumors were removed at an endpoint of 20 mm in diameter and frozen in O.C.T.

### Immunohistochemical Analyses

To visualize protein localization within subcutaneous and primary tumors, tumors were frozen in O.C.T and then sectioned and fixed with 4% paraformaldehyde (PFA) for immunostaining. To visualize protein localization in lung metastases, mice carrying metastases were perfused with 4% PFA and lungs were resected, embedded in O.C.T., and then sectioned for immunostaining. Proteins were detected using mouse anti-vimentin (clone V9, M0725, Dako), rabbit anti-GPR56 (17), rabbit anti-fibronectin (a gift from Dr. Richard Hynes), mouse anti-fibronectin (K094, Sigma), rabbit anti-human nuclei marker (NuMa) (Ab97585, Abcam), rabbit anti-p-FAK (#700255, Invitrogen), mouse anti-human nuclei (huNu) (MAB1281, Millipore), and rabbit anti-pH3 antibodies (#06-570, Millipore), followed by Alexa 488 or 594-conjugated secondary antibodies. Images were acquired using Axio Imager M2m (Zeiss).

Quantitation of immunostaining was performed in ImageJ. Lines were drawn around the borders of each metastatic lesion (as defined by the staining of human-specific antibodies anti-NuMa or anti-huNu) and values were acquired using the Measure tool. At least five control and five GPR56KD metastases were randomly chosen and measured. The mean fluorescence for each metastatic lesion was averaged using Microsoft Excel and analyzed using the Student’s *t*-test.

### Metastasis Assay

Equal numbers of control or GPR56KD cells were injected into NSG mice intravenously through the tail veins. Except for WM983BR cells, which were injected into *Rag2*^−/−^ mice at 1 × 10^6^ per mouse, the remaining human cell lines were injected into NSG mice. 50,000 cells were injected per mouse for MC-1 cells and 451Lu-R cells, 30,000 cells per mouse for MeWo cells, and 20,000 cells per mouse for SK-MEL-147 cells. Mice were euthanized at various time points and lungs were resected, embedded in paraffin or O.C.T., and sectioned. Metastases were detected using the human-specific mouse anti-vimentin antibody, following reported protocol (17, 24, 25).

### Cell Adhesion Assay

MC-1, 451LuR, and MeWo cells expressing EV or GPR56, or their knockdown cells were grown in serum-containing medium. 1 × 10^6^ cells were plated for MC-1 and 451LuR cells, and 2 × 10^6^ cells were plated for MeWo cells. Cells were grown for 2 days and harvested by trypsinization, then resuspended in serum-free medium and counted. 96-well plates were coated with 1, 5, or 10 μg/ml fibronectin (FC010, Millipore) and 1% BSA (BP1600, Fisher) at 37°C for 1 h, then blocked with 1% BSA at 37°C for 1 h to prevent non-specific cell binding. The cells were seeded in the 96-well plates in triplicate and incubated for various lengths of time to allow for adhesion, then non-adherent cells were removed by rinsing with PBS. Remaining adherent cells were fixed with 4% PFA and stained by 1% crystal violet. Cell density was quantitated by extracting crystal violet with 10% acetic acid, and measuring the absorbance at 560 nm. Triplicate absorbance values were averaged. Cells seeded on 1% BSA were used as a negative control, and the average absorbance from fibronectin-coated wells was normalized by subtracting the absorbance from the BSA-coated wells. Mean, SD, and Student’s *t*-test calculations were performed using Microsoft Excel.

### *In Vivo* Treatment with Focal Adhesion Kinase (FAK) Inhibitor

A total of 5 × 10^4^ of MC-1(GPR56KD) cells were injected into the tail vein of 8-week-old male NSG mice (*n* = 10). One week later, the FAK inhibitor Y15 (SML0837, Sigma; 30 mg/kg) or PBS control was administered into these mice daily *via* intraperitoneal injection for 5 days per week for 2 weeks, following reported protocols (26). After treatment, mice were perfused with 4% PFA and lungs were frozen in O.C.T. and sectioned. 10-μm sections were obtained, with a gap of 50 μm between two adjacent sections to avoid redundancies. Metastases were detected by immunohistochemical analyses using the human-specific mouse anti-vimentin antibody and rabbit anti-pH3 antibody. Metastases were scored by the number of vimentin-positive cell clusters per lung section, and their size was determined by the number of vimentin-positive cells in each cluster. Proliferation state of the metastatic lesion was determined by the presence or absence of pH3-positive cells. A total of 30 sections from PBS-treated mice and 30 sections from Y15-treated mice were scored. Statistical analyses were performed using Microsoft Excel. The total number of metastases was determined by the average number of metastases in each lung section. The size of the metastatic lesions was analyzed using the number of lesions with more or less than five cells in each lung section. The number of proliferating metastases in a section was divided by the number of non-proliferating metastases on the same section and graphed as the ratio of proliferating and non-proliferating metastases. The Student’s *t*-test was performed to compare data from PBS- and Y15-treated mice.

## Results

### GPR56 Inhibits the Expansion of Micrometastases to Macrometastases from Melanoma Cell Lines

**Figure 1:**
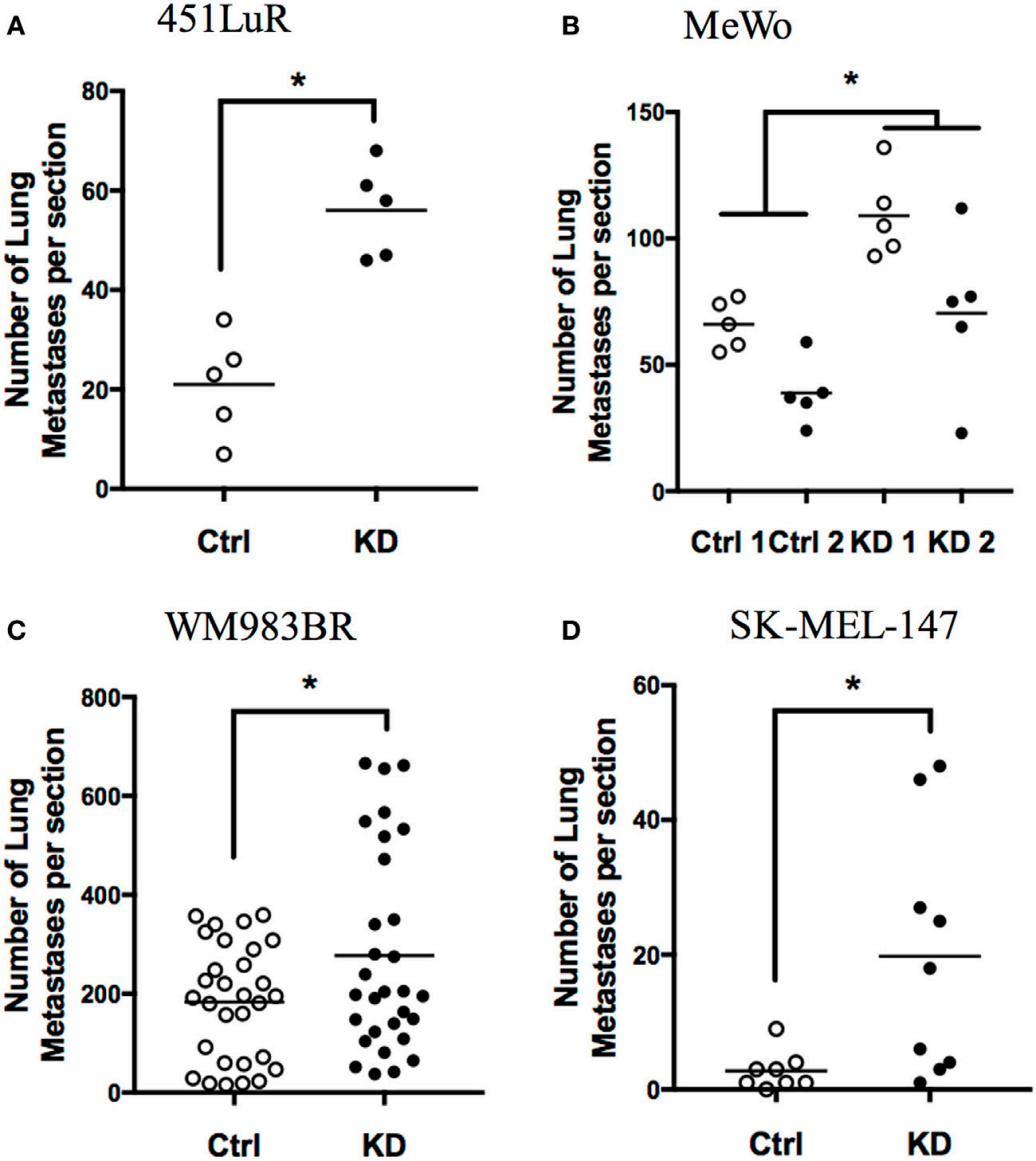
GPR56 inhibits metastatic growth of melanoma cell lines. **(A)** Number of lung metastases from 451LuR cells expressing control-shRNA and GPR56-shRNA (GPR56KD). *p < 0.05. **(B)** Number of lung metastases from MeWo cells expressing two different control-shRNAs and two different GPR56-shRNAs. *p < 0.05. **(C)** Number of lung metastases from WM983BR cells expressing control-shRNA and GPR56-shRNA. *p < 0.05. **(D)** Number of lung metastases from SK-MEL-147 cells expressing control-shRNA and GPR56-shRNA. *p < 0.05.

We previously reported that GPR56 was downregulated in highly metastatic melanoma cells (17), and this downregulation correlates with melanoma malignancy in humans (27, 28). To test the effects of GPR56 on melanoma metastasis, we utilized the experimental metastasis assay and intravenously injected melanoma cells into mice to form lung metastases. We reported that GPR56 expression inhibited melanoma metastasis from the human melanoma cell line, MC-1 (17). Melanomas are known for their heterogeneity and resistance to therapies. ~60% of melanomas express the constitutively active BRAF (BRAF^CA^) and are sensitive to inhibition by BRAF^CA^-specific inhibitors. The BRAF^CA^-specific inhibitors have shown impressive beneficial effects on melanoma treatment but relapse occurs quickly in treated patients, and they are not effective against the ~40% melanomas that express wild-type BRAF (29). The MC-1 cells used in our previous study express BRAF^CA^ and are sensitive to the BRAF^CA^ inhibitors (30). To evaluate how broadly the function of GPR56 applies in melanoma, we recruited four additional human melanoma cell lines with different mutation status and sensitivities to BRAF^CA^ inhibitors (Table 1) (31–33). Lines expressing control-shRNA or GPR56 knockdown (GPR56KD) (Figure S1 in Supplementary Material) were injected into immunodeficient mice, and lungs were resected and analyzed for metastatic growth. A significant increase in lung metastases was observed in the knockdowns of all four cell lines (Figures 1A–D), demonstrating that GPR56 expression inhibits metastatic growth. These four cell lines have different genetic backgrounds, so the inhibition of metastatic growth by GPR56 in these lines suggests a shared mechanism of regulation in melanoma malignancy.

**Table 1:** BRAF and NRAS mutation status and BRAF inhibitor sensitivity of melanoma cell lines.

Cell line	BRAF	NRAS	BRAF inhibitor
MC-1	V600E	WT	Sensitive
MeWo	WT	WT	Resistant
451LuR	V600E	WT	Resistant
WM983BR	V600E	WT	Resistant
SK-MEL-147	WT	Q61R	Resistant

**Figure 2:**
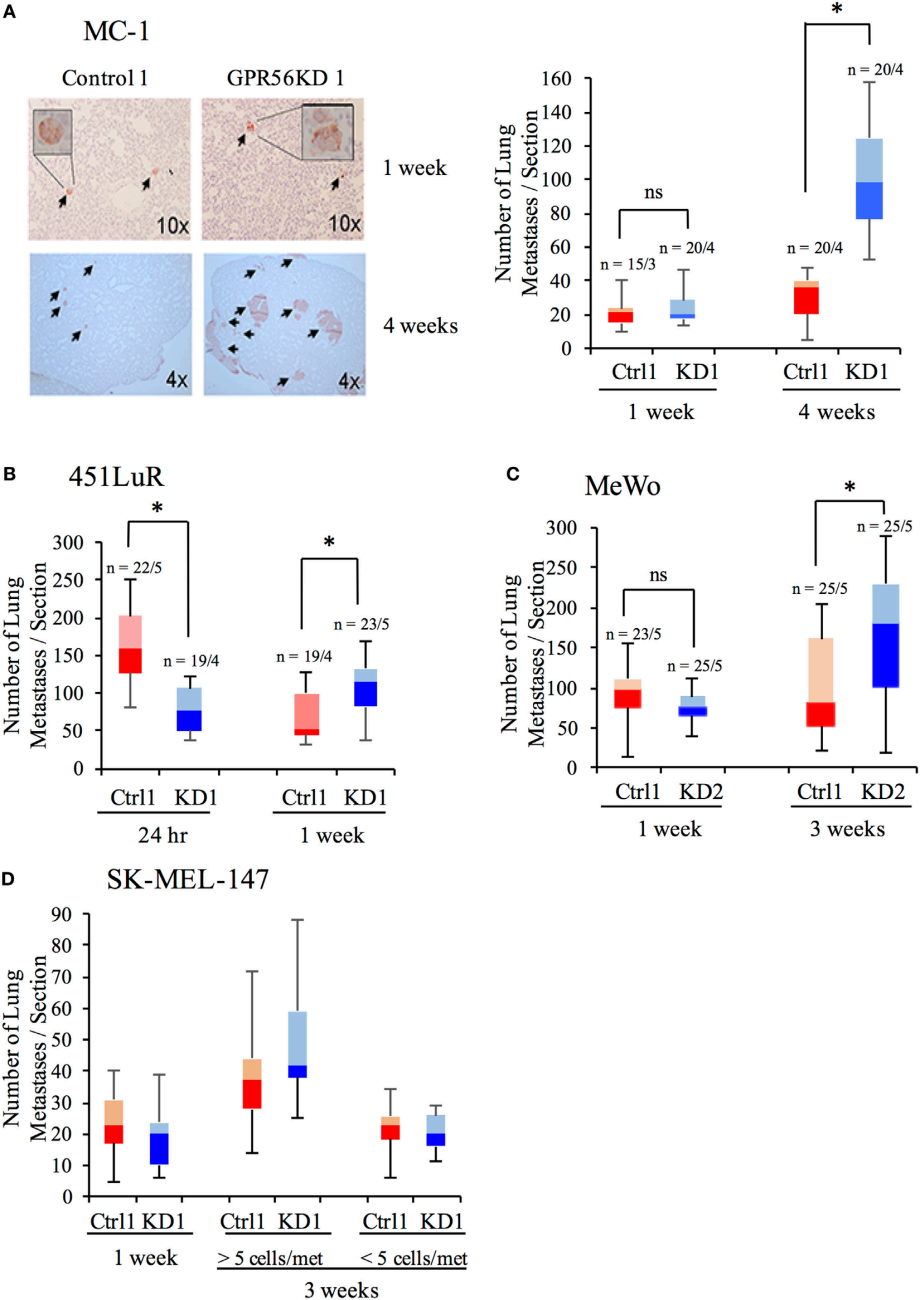
GPR56 inhibits late-stage metastatic growth. **(A)** Number of lung metastases from MC-1(control-shRNA) or MC-1(GPR56KD) cells at 1 or 4 weeks after tail-vein injection. Left: lung sections stained with anti-vimentin antibody to mark human cells. Right: Quantitation of metastases per lung section. *p < 0.05. **(B)** Number of lung metastases from 451LuR(control-shRNA) or 451LuR(GPR56KD) cells at 24 h or 1 week after tail-vein injection. *p < 0.05. **(C)** Number of lung metastases from MeWo(control-shRNA) or MeWo(GPR56KD) cells at 1 or 3 weeks after tail-vein injection. *p < 0.05. **(D)** Number of lung metastases from SK-MEL-147(control-shRNA) or SK-MEL-147(GPR56KD) cells at 1 or 3 weeks after tail-vein injection. During quantitation, metastases at week 3 were separated into two categories based on their size. *p < 0.05.

We next assessed the step at which GPR56 inhibits metastatic growth. During metastatic growth, disseminated cells first proliferate to form small tumors called micrometastases. Once these tumors reach a certain size, they undergo angiogenesis, allowing them to grow into larger tumors called macrometastases (1, 34, 35). Inhibition of either step would result in diminished metastatic growth. We performed time-course assays on four melanoma cell lines—MC-1, 451LuR, MeWo, and SK-MEL-147 (Table 1)—to elucidate the step at which GPR56 acts to inhibit metastatic growth. Control-shRNA and GPR56KD lines were injected into mice and lungs were resected at various time points. Lung sections were stained with human-specific vimentin antibody to detect metastases (Figure 2A, left). Although the cell lines exhibited different growth dynamics, we observed a similar trend among all tested melanoma lines. Depletion of GPR56 did not increase the number of early metastatic lesions (Figures 2A–D; 1 week for A, C, D, and 24 h for B), indicating that it does not inhibit seeding or formation of micrometastases in the lung. However, at later time points, the GPR56KD cells form significantly more metastases than controls (Figures 2A–D; 4 weeks for A, 1 week for B, and 3 weeks for C and D). These later effects suggest that GPR56 inhibits the expansion of lung micrometastases to macrometastases from melanoma cells.

### GPR56 Downregulates Fibronectin Deposition in Subcutaneous Tumors

**Figure 3:**
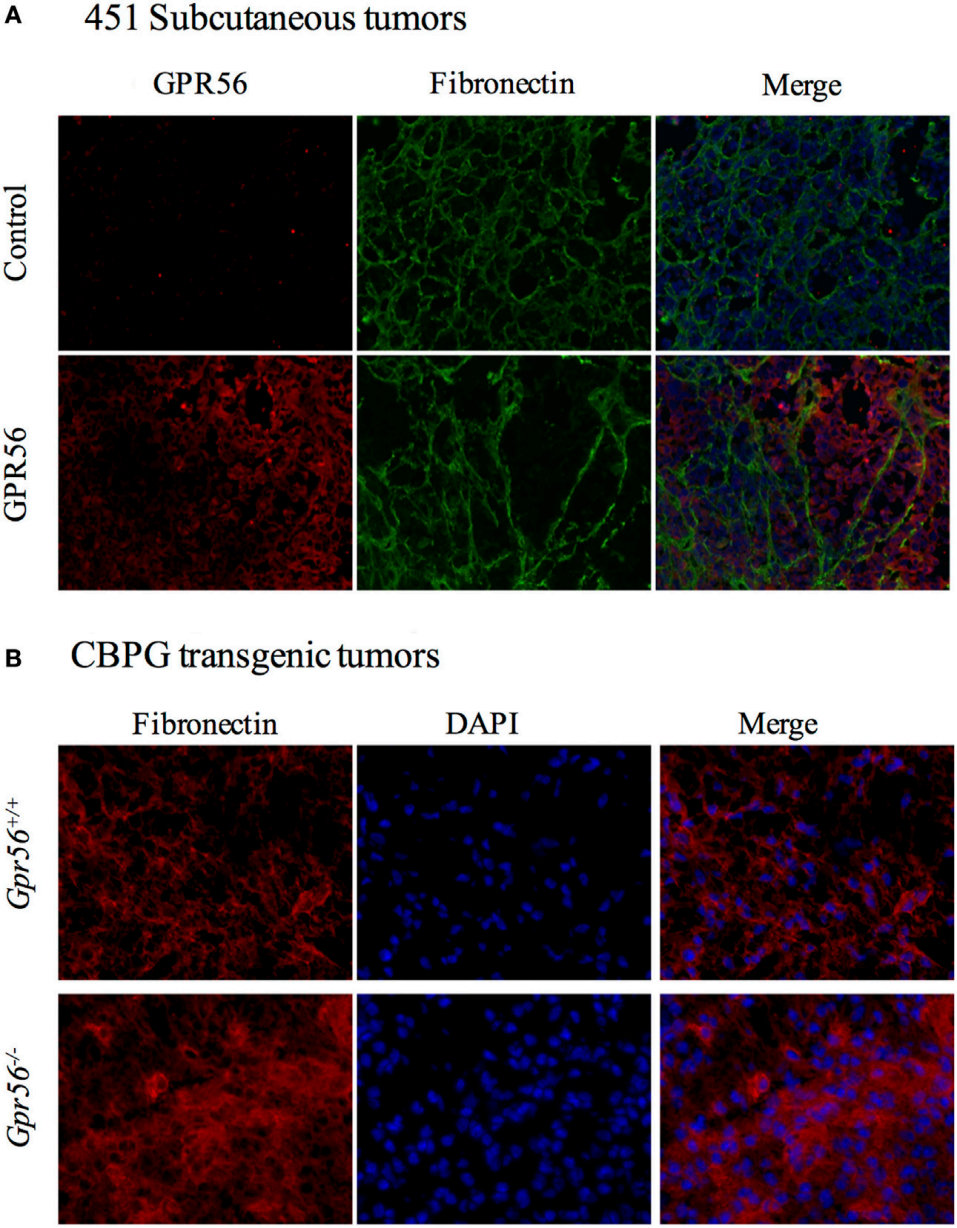
GPR56 inhibits fibronectin deposition in subcutaneous melanomas. **(A)** Changes in fibronectin distribution pattern in tumors expressing GPR56-shRNA (GPR56KD). Tumor sections from 451LuR(control-shRNA) or 451LuR(GPR56KD) cells were stained with the anti-GPR56 antibody (red) and the mouse anti-fibronectin antibody (green). Blue: DAPI, stained for nuclei. **(B)** Changes in fibronectin distribution pattern in tumors from *GPR56^−/−^* mice. Spontaneous melanomas from *Tyr:CreER*; *BRaf^CA^*; *Pten^lox/lox^* and *Tyr:CreER*; *BRaf^CA/+^*; *Pten^lox/lox^; Gpr56^−/−^*(CBPG) mice were sectioned and stained with the rabbit anti-fibronectin antibody (red). Blue: DAPI, stained for nuclei.

Extracellular matrix is a major component of the tumor microenvironment, and it promotes tumor growth by providing growth and survival signals to cancer cells (9, 36). Our lab has previously reported that GPR56 expression inhibited deposition of fibronectin, an important ECM protein, in xenograft tumors from the human melanoma cell line MC-1 (18). We sought to examine whether this relationship is applicable to other melanoma cell lines. GPR56 was overexpressed in 451LuR cells (Table 1), which were injected subcutaneously into mice. Fibronectin content was analyzed by immunostaining in the tumors (see Materials and Methods). Expression of GPR56 induced a “fishnet” distribution pattern of fibronectin in 451LuR tumors (Figure 3A), similar to the pattern previously observed in MC-1 tumors (18). Within the GPR56 overexpressing tumors, higher GPR56 levels associated with larger gaps in fibronectin staining (Figure 3A), consistent with a local downregulation of fibronectin by GPR56.

A transgenic mouse model was also used to determine whether the inhibition of fibronectin deposition by GPR56 existed in spontaneous tumors. Xenograft models are valuable tools for studying tumor growth, but do not recapitulate the spontaneous tumors formed in humans. Transgenic mouse models allow for the generation of spontaneous tumors by expressing temporally controlled oncogenes in specific cell types. Furthermore, xenograft experiments are performed in immunodeficient mice whereas transgenic mice have intact immune systems, which can affect the dynamics of tumor growth. The transgenic CBP mice form spontaneous melanomas in response to 4-hydroxytamoxifen exposure, and their melanoma development recapitulates primary tumor growth in humans (19). We crossed these mice with *Gpr56^−/−^* mice to examine the effects of GPR56 loss on ECM content in these tumors (19, 20). Tumors from *Gpr56^−/−^* mice were sectioned and stained with the anti-fibronectin antibody. A higher level of fibronectin was observed in these tumors, relative to those from *Gpr56^+/+^* mice (Figure 3B), suggesting that GPR56 also inhibits fibronectin deposition in spontaneously arisen melanomas from immunocompetent mice.

### Fibronectin Deposition in Metastases Is Inhibited by GPR56 Expression

**Figure 4:**
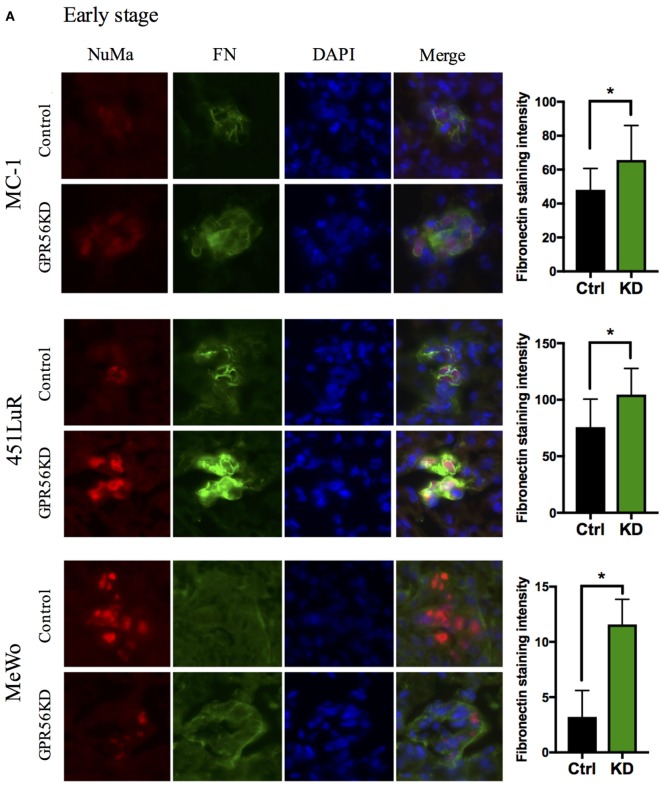

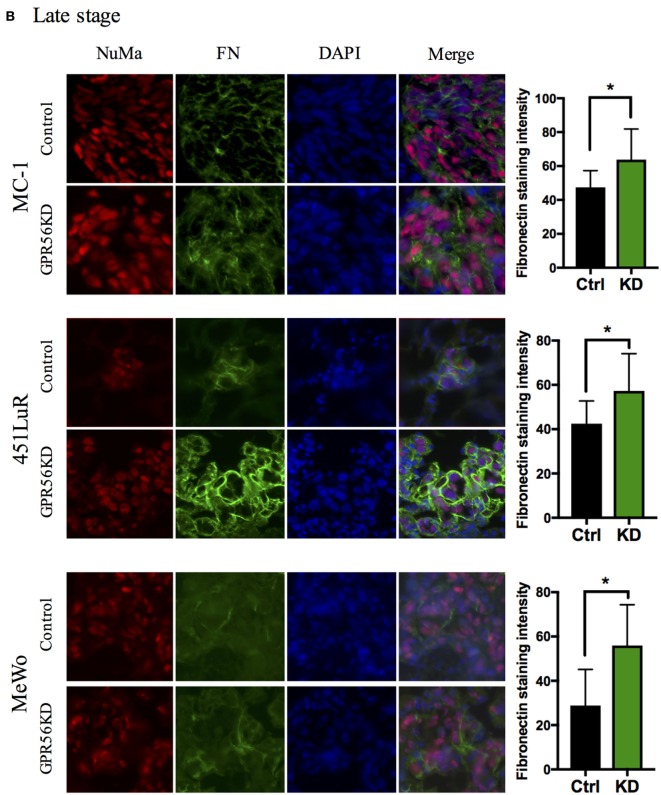
Depletion of GPR56 leads to increased fibronectin deposition in early- and late-stage metastases. **(A)** Increased fibronectin was observed in early-stage metastases depleted of GPR56. Left: lung sections from mice injected with MC-1, 451LuR, or MeWo cells expressing control-shRNA and GPR56-shRNA were stained with the mouse anti-fibronectin antibody (green). Red: human-specific anti-nuclei marker (NuMa) antibody to label human cells. Blue: DAPI, for nuclei. Right: quantitation of fibronectin staining intensity. The intensity was measured using the ImageJ software (see Materials and Methods). **(B)** Increased fibronectin was observed in late-stage metastases depleted of GPR56. Left: lung sections from mice injected with MC-1, 451LuR, and MeWo cells expressing control-shRNA and GPR56-shRNA were stained with the mouse anti-fibronectin antibody (green). Red: human-specific anti-NuMa antibody to label human cells. Blue: DAPI, for nuclei. Right: quantitation of fibronectin staining intensity. The intensity was measured using the ImageJ software (see Materials and Methods).

Due to the above effects of GPR56 on fibronectin content in sub-cutaneous tumors, we hypothesized that GPR56 inhibits fibro-nectin deposition in melanoma metastases, resulting in impaired metastatic growth. If this is the case, we would observe differences in fibronectin levels in control and GPR56KD metastases, perhaps before differences in metastatic growth become apparent. To test this, early- and late-stage metastases from different melanoma cell lines were analyzed for fibronectin content by immunostaining using an antibody specific to human fibronectin (see Materials and Methods). Metastases with GPR56 knockdown deposited more fibronectin than control metastases in all the cell lines tested (Figure 4). This difference was found in early-stage metastases, before a difference in size or number of metastases was observed (Figure 4A), and persisted into later stages (Figure 4B). These results support our prediction that GPR56 inhibits fibronectin deposition in the metastatic microenvironment, which may lead to inhibition on metastatic growth.

### Expression of GPR56 Inhibits Adhesion of Melanoma Cells on Fibronectin and Its Downstream Signaling

**Figure 5:**
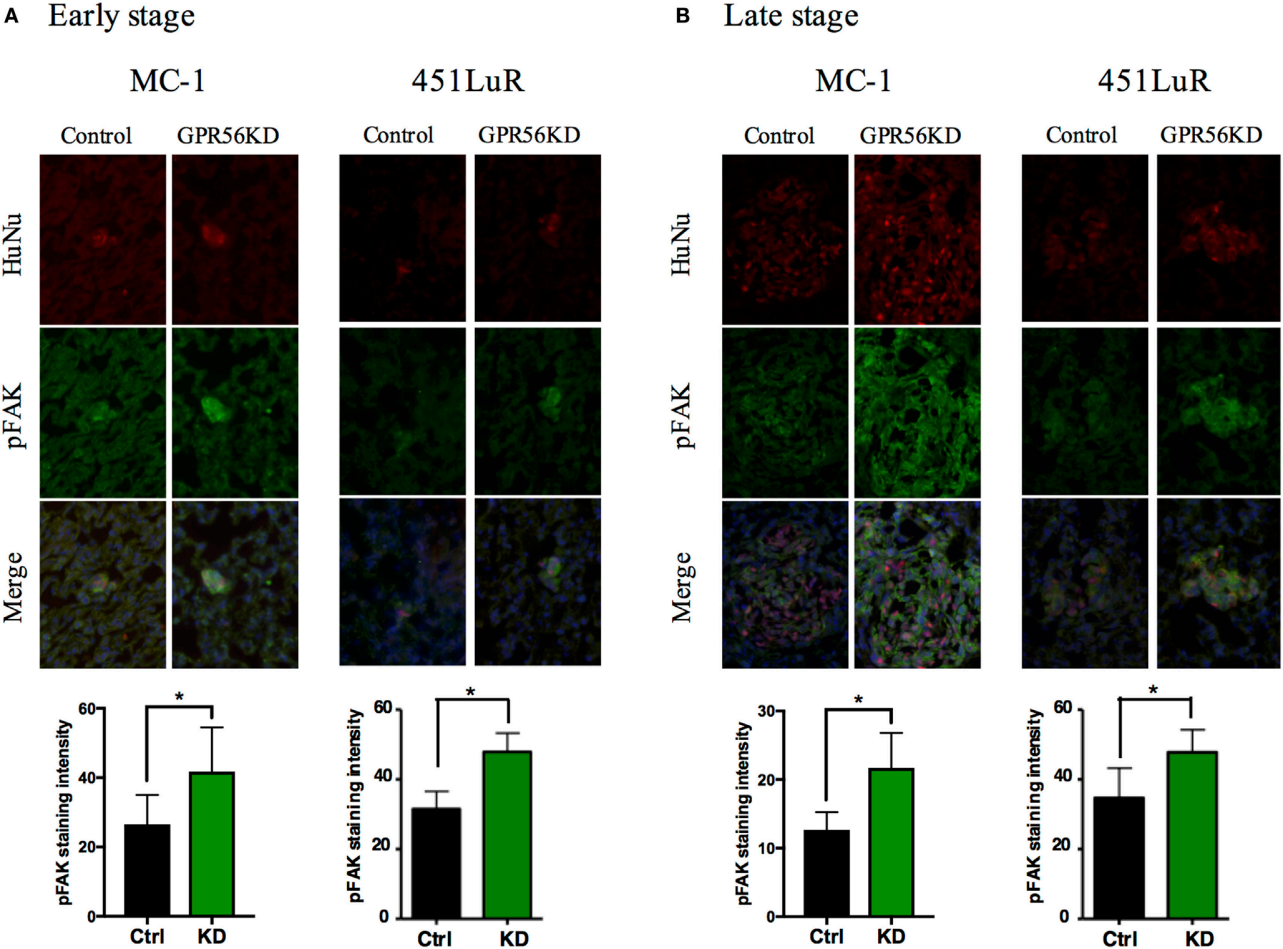
Cell–extracellular matrix signaling is inhibited by GPR56 expression *in vivo*. **(A)** GPR56 depletion leads to increased p-FAK staining in 1-week metastases. Top: lung sections from mice injected with MC-1 or 451LuR cells expressing control-shRNA and GPR56-shRNA were sectioned and stained with the anti-p-FAK antibody (green). Red: human-specific anti-human nuclei (huNu) antibody to label human cells. Blue: DAPI, for nuclei. Bottom: quantitation of p-FAK staining intensity. The intensity was measured using the ImageJ software (see Materials and Methods). **(B)** GPR56 depletion leads to increased p-FAK staining in 4-week (MC-1) and 3-week (451LuR) metastases. Top: lung sections from mice injected with MC-1 or 451LuR cells expressing control-shRNA and GPR56-shRNA were sectioned and stained with the anti-p-FAK antibody (green). Red: human-specific anti-huNu antibody to label human cells. Blue: DAPI, for nuclei. Bottom: quantitation of p-FAK staining intensity. The intensity was measured using the ImageJ software (see Materials and Methods).

Cells bind to ECM proteins *via* adhesion receptors and form focal adhesion complexes, which transmit signals between the cell and the microenvironment (37–39). This signaling is critical for cell growth and survival, so upregulation of cell–ECM signaling would be expected to promote metastatic growth (40, 41). Our previous and current findings on the connection between GPR56 and cell–ECM signaling indicate that GPR56 might inhibit melanoma metastasis *via* blocking cell–ECM interactions (18, 42–44). One of the major downstream effectors of cell–ECM signaling is FAK (45). FAK is upregulated in many cancers, and our lab has previously shown that high levels of GPR56 are sufficient to cause downregulation of FAK in subcutaneous melanomas (18, 42–44). To determine whether GPR56 also inhibits cell–ECM signaling in metastases, we analyzed FAK activity in control and GPR56KD lung metastases from MC-1 and 451LuR cells, using the antibody against phospho-FAK (the active form of FAK). We observed elevated FAK activation in GPR56KD metastases at 1 week (Figure 5A), before differences in metastatic growth were observed, and it persisted at later stages (Figure 5B). These results indicate that GPR56 inhibits the downstream signaling from cell–ECM adhesion in early metastatic lesions, which might lead to the impaired metastatic growth at later time points.

**Figure 6:**
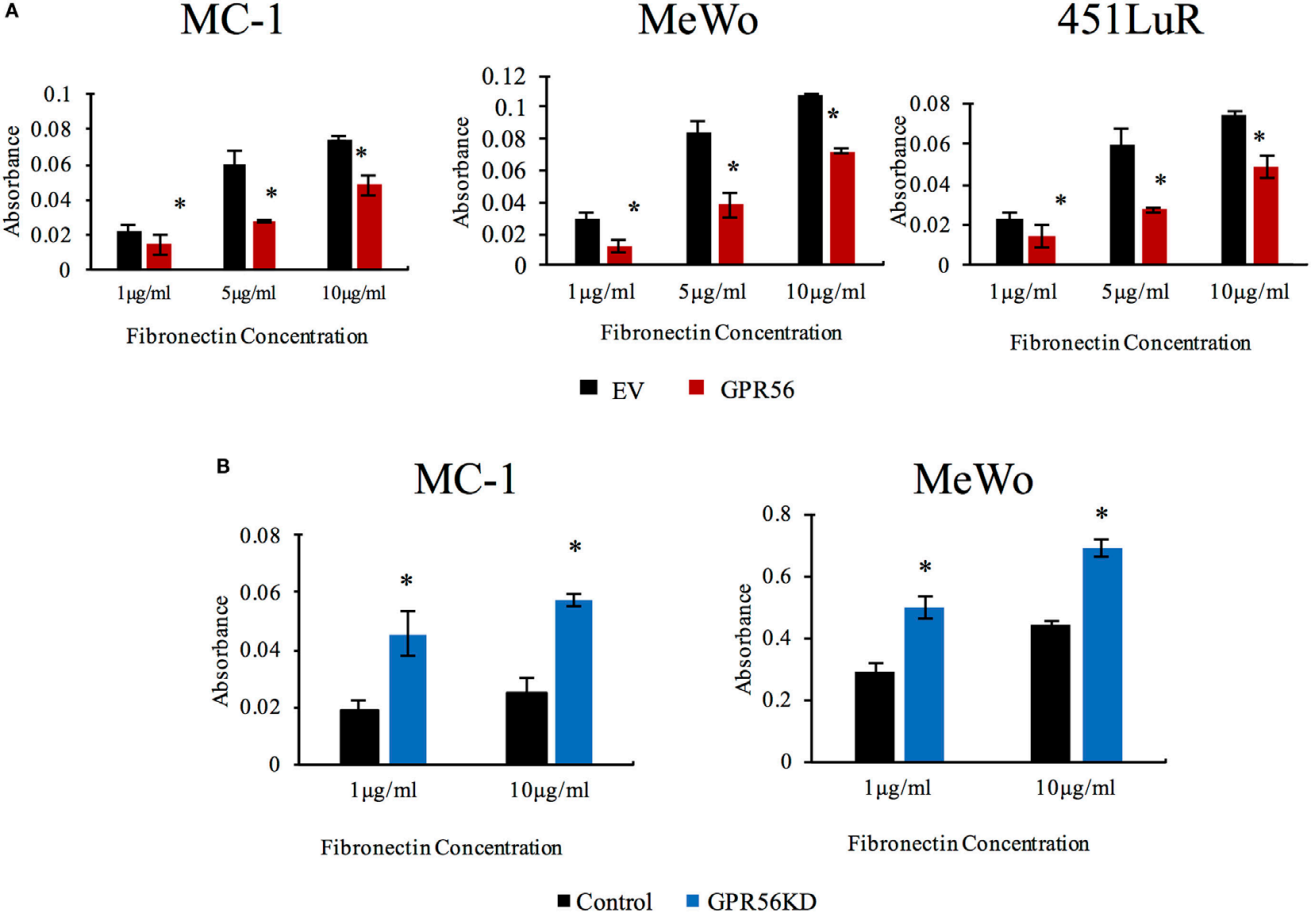
GPR56 expression inhibits cell adhesion to fibronectin. **(A)** Over-expression of GPR56 inhibits cell adhesion to fibronectin. Adhesion of MC-1, 451LuR, or MeWo cells expressing empty vector (EV) or GPR56 to plates coated with 1, 5, and 10 μg/ml fibronectin. Adhesion was quantitated by crystal violet staining and measuring the absorbance of the elution. *p < 0.05. **(B)** Depletion of GPR56 elevates cell adhesion to fibronectin. Adhesion of MC-1 or MeWo cells expressing control-shRNA or GPR56KD to plates coated with 1 and 10 μg/ml fibronectin. Adhesion was quantitated by crystal violet staining and measuring the absorbance of the elution. *p < 0.05.

The inhibition of FAK activity and fibronectin deposition in melanoma metastasis by GPR56 led us to predict that GPR56 inhibits melanoma cell adhesion on fibronectin. To test this, MC-1, 451LuR, and MeWo cells overexpressing GPR56, or GPR56KD, and their respective controls were plated on various amounts of fibronectin *in vitro* and assessed for their adhesion ability. We observed that overexpression of GPR56 inhibited cell adhesion to fibronectin in all three cell lines (Figure 6A), and GPR56KD increased cell adhesion to fibronectin (Figure 6B). These findings support that GPR56 inhibits cell–fibronectin adhesion.

### Activation of FAK Is Required for the Proliferation of Lung Metastases from Melanoma Cells

GPR56 is downregulated in metastatic cells, and we have shown that this downregulation leads to elevated cell–ECM signaling and cell adhesion. However, it is unclear if this regulation is required for its role in inhibiting metastatic growth. GPR56 may have multiple downstream effects on melanoma cells apart from its inhibition on cell–ECM signaling, so we sought to directly determine if the cell–ECM signaling inhibition by GPR56 is causative for its impediment on lung metastases. The small molecule FAK inhibitor Y15 was utilized in the experimental metastasis assay to test this (26, 46, 47). We reasoned that, if elevated FAK signaling is responsible for the increased expansion of micrometastases to macrometastases upon GPR56KD, loss of FAK activation upon Y15 treatment should reverse this.

**Figure 7:**
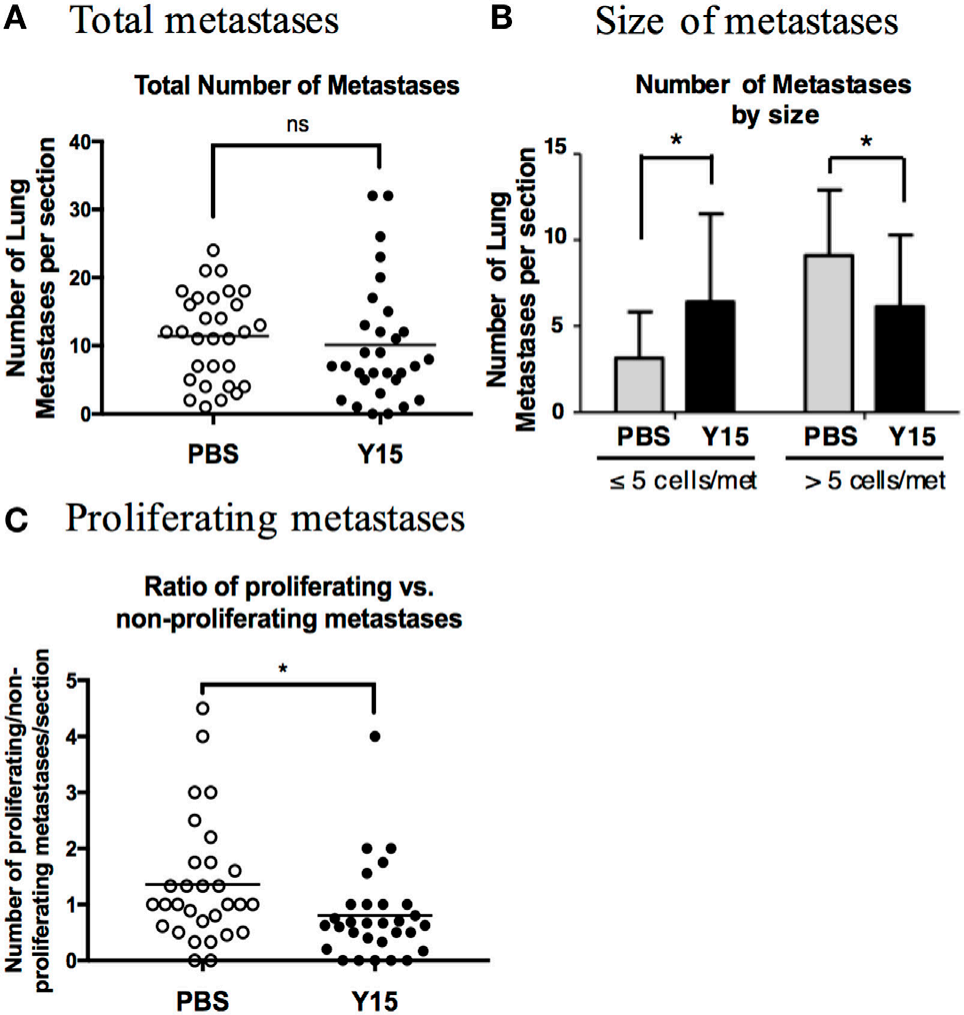
Focal adhesion kinase (FAK) inhibition blocked proliferation in metastases. **(A)** Total number of metastases per lung section. Mice were injected with MC-1(GPR56KD) cells and treated with PBS control or Y15 FAK inhibitor, and lung sections were stained with anti-vimentin antibody to visualize metastases. ns: not significant. **(B)** Number of metastases per lung section with ≤5 cells or >5 cells. Mice were injected with MC-1(GPR56KD) cells and treated with PBS control or Y15 FAK inhibitor, and lung sections were stained with anti-vimentin antibody to visualize metastases. Metastases were quantitated based on the number of vimentin-positive cells in each cell cluster. *p < 0.05. **(C)** Ratio of proliferating to non-proliferating metastases after PBS or Y15 treatment. Sections of lungs treated with PBS or Y15 were stained with anti-vimentin antibody and anti-p-H3 antibody, and vimentin-positive metastases with one or more p-H3 positive cells were graded as proliferative. *p < 0.05.

MC-1(GPR56KD cells) were injected into mice and allowed to grow for 1 week to establish lung micrometastases. Mice were treated with PBS or Y15 for 2 weeks, and lungs were resected to analyze metastatic growth. We counted the metastases in each lung section and observed no significant difference in the total number of metastases between PBS- and Y15-treated mice (Figure 7A). This suggests that inhibiting cell–ECM signaling after the formation of micrometastases is not sufficient to completely reverse the growth advantage imposed by GPR56KD. However, when we quantitated the metastases based on their size, we found that Y15 treatment led to smaller lung metastases than PBS treatment. Y15-treated mice had significantly more small metastases, whereas PBS-treated mice had significantly more large metastases (Figure 7B). Therefore, although FAK inhibition does not reduce the total number of lung metastases, it is sufficient to sequester them at a micrometastatic state. To confirm this difference in growth, we stained the lung sections with the anti-p-H3 antibody to mark cells in the metaphase stage of mitosis. We found that the portion of proliferating metastases over total metastases was significantly lower in mice treated with Y15 (Figure 7C). Consequently, cell adhesion signaling is required for the proliferation and expansion of lung metastases, and its inhibition by GPR56 may contribute to its negative effects on metastasis formation from melanoma cells.

## Discussion

Metastatic melanoma is a deadly disease that is resistant to many current therapies, and new methods are needed to combat its progression (48–50). ECM protein accumulation and aberrant cell–ECM signaling have been implicated in the growth of several cancers, so reversing these processes may represent novel therapeutics for cancer treatment (5, 10, 51). However, regulatory mechanisms of ECM accumulation are poorly understood and approaches for reversal are not yet available.

We reported previously that the adhesion GPCR, GPR56, inhibited the metastasis of a melanoma cell line and its expression negatively correlates with melanoma malignancy (17). We show here that it inhibits the metastatic growth of four additional melanoma cell lines with different genetic mutations, confirming the importance of GPR56 in inhibiting melanoma metastasis. Time course analyses were performed to determine the step at which GPR56 inhibits metastatic growth. GPR56 did not affect the formation of lung micrometastases, but inhibited the expansion of micrometastases into macrometastases. Micrometastases are small metastatic tumors that are not clinically detectable, and diagnostic methods are needed to identify these tumors early to begin treatment before the disease spreads further (2, 35). If GPR56 expression sequesters lung metastases at a micrometastatic state, then it has potential as an early indicator of metastatic disease. Furthermore, GPR56 is downregulated in highly metastatic melanoma cells compared to poorly metastatic cells (17), so expression levels in metastases may also serve as a prognostic marker for the metastatic potential of the tumor.

Our previous studies indicated that GPR56 regulates fibronectin content in xenograft tumors, and due to the role of ECM in cancer growth and metastasis we predicted that GPR56 might inhibit metastatic growth through its effects on ECM (18). We found that high fibronectin levels correlated with low GPR56 expression in xenograft and spontaneous primary melanomas, as well as in metastases. In some cases, fibronectin content was elevated in GPR56KD cells even before differences in number of metastases became apparent. This indicated that alterations in ECM deposition are an early-stage effect of GPR56 depletion.

Cell–ECM signaling is important in cancer cell proliferation and survival and is activated by cell surface receptors binding to ECM proteins (5, 9). We predicted that increased fibronectin in GPR56 knockdown metastases might lead to increased cell–ECM signaling and, therefore, an enhancement in growth. Consistent with this prediction, higher p-FAK levels were found in GPR56 knockdown metastases relative to the controls and GPR56 expression was sufficient to inhibit cell adhesion on fibronectin *in vitro*. Furthermore, treatment with the FAK inhibitor, Y15, inhibited the proliferation of GPR56KD metastases, suggesting that FAK activation, and thus cell–ECM signaling, is required for the growth advantage of GPR56KD cells in lung metastases.

Focal adhesion kinase has been widely implicated in cancer progression, and thus has been proposed as a potential therapeutic target and its inhibitors were studied in clinical trials (42, 44, 47). Our study confirmed the efficacy of FAK inhibition on metastatic proliferation *in vivo*, but it also revealed that FAK inhibition was not sufficient to reduce the total number of metastases. Furthermore, FAK is a major kinase that regulates numerous cellular functions such as proliferation, cell survival, and gene expression, so its inhibition would have widespread effects apart from cell adhesion and its inhibition may have detrimental side effects in cancer patients (45). Finally, as evidenced by reduced cell proliferation upon FAK inhibition, inhibition of cell–ECM signaling may contribute to the effects of GPR56 on melanoma metastasis, but it cannot be the only mechanism of regulation. GPR56 expression, but not FAK inhibition, is sufficient to reduce the number of lung metastases, so other downstream effects of GPR56 must be involved in its inhibition of metastatic growth.

GPR56 has been shown to inhibit mesenchymal differentiation in glioblastoma, implicating it as a potential negative regulator of epithelial to mesenchymal transition (EMT) (52). In EMT, cells lose polarity and cell–cell adhesion and gain a mesenchymal phenotype (53–55), and EMT plays a crucial role in metastasis by promoting cell migration and invasion behavior (55, 56). If GPR56 inhibits EMT, then this may be a mechanism by which it inhibits metastatic growth. Indeed, fibronectin is considered a mesenchymal marker of EMT, and we show here that GPR56 depletion is sufficient to increase fibronectin deposition in melanoma metastases (54). The regulation of EMT by GPR56 could explain why FAK inhibition alone was not sufficient to inhibit metastatic growth in our model.

The mechanism by which GPR56 regulates ECM deposition and cell adhesion signaling needs to be investigated further. GPR56 may regulate adhesion *via* internalization of ECM proteins during its endocytosis from the cell membrane or through GPCR activity and downstream signaling. Our lab published previously that GPR56 binds and internalizes transglutaminase 2 (TG2) from the ECM, leading to its degradation in lysosomes (18). TG2 is a major ECM crosslinking enzyme, and its removal from the ECM may lead to destabilization or depletion of other ECM proteins, such as fibronectin (57–59). This loss of fibronectin may lead to reduced cell adhesion. Alternatively, GPR56 may inhibit cell–ECM signaling through other mechanisms. GPR56 has been shown to activate RhoA in oligodendrocyte cells and to inhibit the activation of PKCα in melanocytes (27, 60). Both of these signaling proteins have roles in focal adhesion complex formation and may represent a mechanism of regulating cell adhesion (61, 62). Furthermore, GPR56 signaling may affect expression of cell adhesion and ECM regulatory proteins such as matrix metalloproteinases (MMPs). MMPs have complex tumor promoting and inhibitory activities, and their altered expression could affect fibronectin content and metastatic progression (63, 64).

GPR56 is a member of the adhesion GPCR family, a unique set of receptors that signal through G proteins but also play roles in cell adhesion and cell–cell and cell–matrix interactions (16, 65). The function of adhesion GPCRs in cancer progression has been reported (66), although the mechanisms of regulation are poorly understood. GPR56 was reported by us and other to be downregulated during melanoma malignancy in humans, and the adhesion GPCRs LPN3 and BAI3 were found frequently mutated in human cancer samples, further strengthening the clinical relevance of these receptors on cancer progression (67). The adhesion GPCRs appear to exert diverse roles in cancer, however, since some were reported to promote cancer progression (68–70), but others inhibit it (17, 18, 27, 71). Further studies on the biochemical and physiological functions of adhesion GPCRs will be needed to properly access their potential as therapeutic targets to combat cancer progression.

## Ethics Statement

This study was carried out in accordance with the recommendations of the animal care guidelines from the Division of Laboratory Animal Medicine at the University of Rochester Medical Center. The protocol was approved by the University Committee on Animal Resources (UCAR) at the University of Rochester Medical Center.

## Author Contributions

Conception and design: MM and LX. Acquisition of data: MM and NC. Analysis and interpretation of data, MM, NC, and LX. Writing, review, and revision of the manuscript: MM and LX. Study supervision: LX.

## Conflict of Interest Statement

The authors declare that the research was conducted in the absence of any commercial or financial relationships that could be construed as a potential conflict of interest.
