# Supplementary material for: The Adhesion G-Protein-Coupled Receptor, GPR56/ADGRG1, Inhibits Cell–Extracellular Matrix Signaling to Prevent Metastatic Melanoma Growth
Source: Front Oncol. 2018 Feb 1;8:8. doi: 10.3389/fonc.2018.00008 (PMC5799216; doi:10.3389/fonc.2018.00008)
Supplement: Supplementary file 1 [file Data_Sheet_1.docx]

**The adhesion GPCR, GPR56/ADGRG1, inhibits cell-ECM signaling to prevent metastatic melanoma growth**

**Michelle W. Millar, Nancy Corson, Lei Xu***

*** Correspondence:** Lei Xu: lei_xu@urmc.rochester.edu


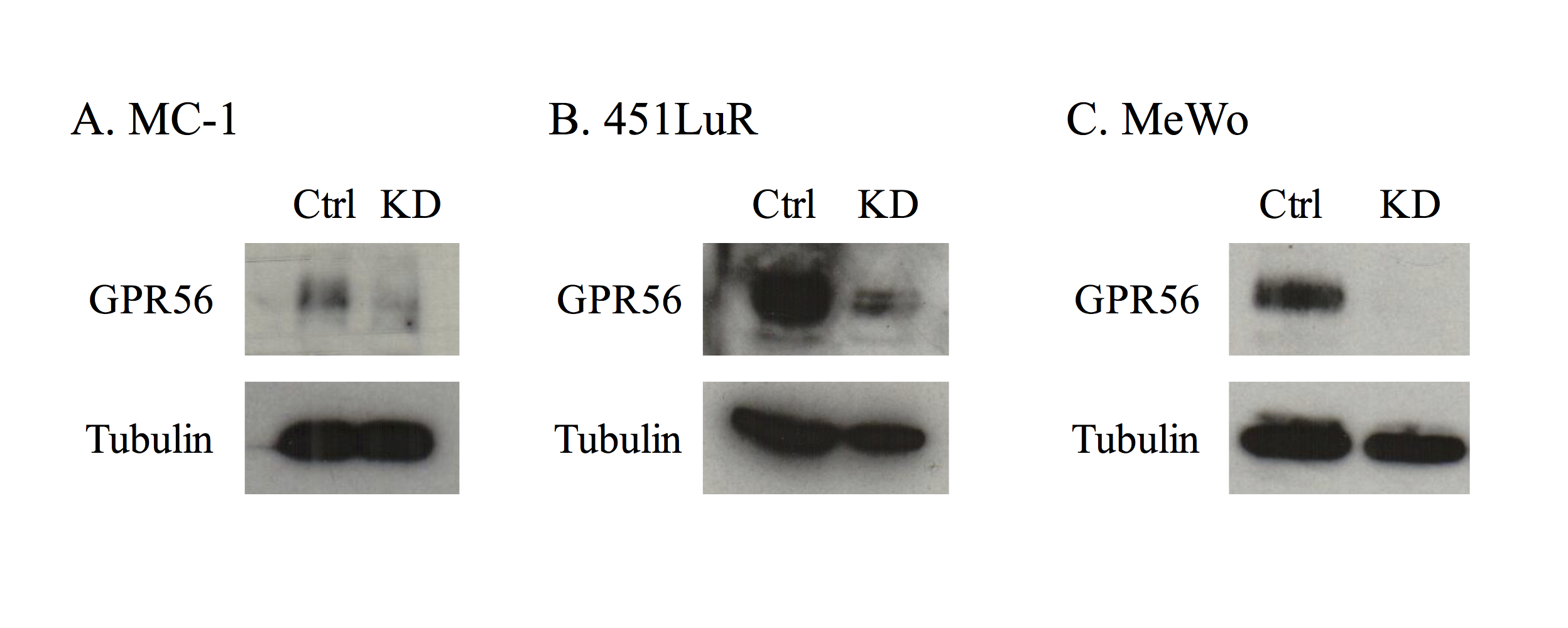


**Supplementary Figure 1.** GPR56 knockdown in cell lines.

**(A-C)** Western blots of lysates from **(A)** MC-1, **(B)** 451LuR, or **(C)** MeWo cells expressing control-shRNA or GPR56-shRNA.
